# Determining the sample size for a cluster-randomised trial using knowledge elicitation: Bayesian hierarchical modelling of the intracluster correlation coefficient

**DOI:** 10.1177/17407745231164569

**Published:** 2023-04-10

**Authors:** Svetlana V Tishkovskaya, Chris J Sutton, Lois H Thomas, Caroline L Watkins

**Affiliations:** 1 Lancashire Clinical Trials Unit, Faculty of Health and Care, University of Central Lancashire, Preston, UK; 2 Centre for Biostatistics, Division of Population Health, Health Services Research & Primary Care, School of Health Sciences, Faculty of Biology, Medicine and Health, The University of Manchester, Manchester, UK; 3 Faculty of Allied Health and Wellbeing, University of Central Lancashire, Preston, UK

**Keywords:** Bayesian hierarchical model, cluster-randomised trial, intracluster correlation coefficient, knowledge elicitation, post-stroke incontinence, sample size determination

## Abstract

**Background:**

The intracluster correlation coefficient is a key input parameter for sample size determination in cluster-randomised trials. Sample size is very sensitive to small differences in the intracluster correlation coefficient, so it is vital to have a robust intracluster correlation coefficient estimate. This is often problematic because either a relevant intracluster correlation coefficient estimate is not available or the available estimate is imprecise due to being based on small-scale studies with low numbers of clusters. Misspecification may lead to an underpowered or inefficiently large and potentially unethical trial.

**Methods:**

We apply a Bayesian approach to produce an intracluster correlation coefficient estimate and hence propose sample size for a planned cluster-randomised trial of the effectiveness of a systematic voiding programme for post-stroke incontinence. A Bayesian hierarchical model is used to combine intracluster correlation coefficient estimates from other relevant trials making use of the wealth of intracluster correlation coefficient information available in published research. We employ knowledge elicitation process to assess the relevance of each intracluster correlation coefficient estimate to the planned trial setting. The team of expert reviewers assigned relevance weights to each study, and each outcome within the study, hence informing parameters of Bayesian modelling. To measure the performance of experts, agreement and reliability methods were applied.

**Results:**

The 34 intracluster correlation coefficient estimates extracted from 16 previously published trials were combined in the Bayesian hierarchical model using aggregated relevance weights elicited from the experts. The intracluster correlation coefficients available from external sources were used to construct a posterior distribution of the targeted intracluster correlation coefficient which was summarised as a posterior median with a 95% credible interval informing researchers about the range of plausible sample size values. The estimated intracluster correlation coefficient determined a sample size of between 450 (25 clusters) and 480 (20 clusters), compared to 500–600 from a classical approach. The use of quantiles, and other parameters, from the estimated posterior distribution is illustrated and the impact on sample size described.

**Conclusion:**

Accounting for uncertainty in an unknown intracluster correlation coefficient, trials can be designed with a more robust sample size. The approach presented provides the possibility of incorporating intracluster correlation coefficients from various cluster-randomised trial settings which can differ from the planned study, with the difference being accounted for in the modelling. By using expert knowledge to elicit relevance weights and synthesising the externally available intracluster correlation coefficient estimates, information is used more efficiently than in a classical approach, where the intracluster correlation coefficient estimates tend to be less robust and overly conservative. The intracluster correlation coefficient estimate constructed is likely to produce a smaller sample size on average than the conventional strategy of choosing a conservative intracluster correlation coefficient estimate. This may therefore result in substantial time and resources savings.

## Background

Health interventions are often evaluated using cluster-randomised trials where clusters of individuals are randomly allocated to trial arms.^1^ Typically, subjects within the same cluster have similar outcomes, not just because they are similar, but as they may share unmeasured cluster-level effects on outcome. The correlation of outcome measurements within a cluster, called the intracluster correlation coefficient (ICC or ρ ),^2,3^ must be utilised in trial design and analysis. The ICC is a most commonly used measure of the similarity of clustered data.^3^ It compares within-group variance $\sigma_{w}^{2}$ with between-group variance $\sigma_{b}^{2}$ and for a continuous outcome is defined as

$$\rho =\frac{\sigma_{b}^{2}}{\sigma_{b}^{2}+\sigma_{w}^{2}}$$

Similarities among individuals within clusters cause a loss of statistical power to detect a between-group difference. To maintain power, the target sample size must be inflated to allow for clustering: the sample size of an individually randomised trial is multiplied by the design effect DEff = 1 + (*m* − 1)×*ρ*, where *m* is the average cluster size. If *m* is large, the inflation factor may substantially increase the sample size, even when *ρ* is small (as it often is in cluster-randomised trials).

As sample size is highly sensitive to the ICC, robust ICC estimates are required. Currently, obtaining a robust estimate of the ICC is a challenge in planning a cluster-randomised trial.^1,3,4^ An underestimated ICC will produce an underpowered study, whereas an inflated ICC will require more clusters and/or participants than necessary, leading to wasted resources and a potentially unethical trial.

The usual, and easiest, way of obtaining an ICC is using a single estimate from an existing source, such as a published similar study, published lists or databases of ICC estimates, or a pilot for the proposed study. Use of a single ICC value is not robust (often resulting in a trial being underpowered or overpowered causing wasted resources) and a sufficiently relevant value is seldom available.^1,4–6^

An advanced approach might use multiple-estimate methods, combining different ICC estimates for the particular outcome and cluster type. However, finding multiple sources highly relevant to the target outcome, population and cluster type is quite difficult. It is likely that available studies with ICCs will have only partial relevance to the planned trial. Furthermore, there is no recommended method of combining ICCs and simple approaches, such as using their mean, median or maximum, do not account for the specific characteristics of the studies, the degree of relevance or the uncertainty in each ICC estimate.

Imprecision in the ICC is usually expressed in terms of variance of the estimate, with different ways of calculating ICC variance proposed.^1^ Power calculations for cluster-randomised trials typically use ICC estimates without taking their uncertainty into account.^7^ Accounting for imprecision in the ICC would improve the estimation of the required sample size and protect against trials having inadequate power due to a higher than expected ICC.

In this study, we adopted and extended the method of combining ICC values in the Bayesian framework^8^ suggested by Turner et al.^9^ who applied the Bayesian hierarchical approach to combine multiple relevant ICC estimates in a single model. The method can be applied to ICCs of varying relevance, thus relaxing the limitations of other approaches which require a high level of similarity across studies. Using this method, the input of each study is differentiated according to its degree of relevance. Relevance to target setting is expressed as weights for each study and for each study outcome. Turner et al.^9^ assigned categorised weights to studies and outcomes according to their relevance. Thus, the method allows incorporation of all available information, including less relevant data sources, into the model, allowing the latter to have less influence when combining ICC estimates.

We extended the approach suggested in Turner et al.^9^ by incorporating expert knowledge elicitation process into the Bayesian modelling to assist in assessing the relevance weights and deciding how much strength can be borrowed from each study. The motivation for developing the methodology presented here was a proposed cluster-randomised trial of the effectiveness of a systematic voiding programme for people on National Health Service (NHS) stroke units with stroke and urinary incontinence. The primary outcome measure was the International Consultation on Incontinence Questionnaire–Urinary Incontinence Short Form (ICIQ-UI-SF) score, assessing incontinence symptom severity.^10^

A robust ICC estimate was not available to inform the proposed trial, although a feasibility trial, Identifying Continence OptioNs after Stroke (ICONS),^11^ had been conducted. ICONS produced an imprecise ICC estimate due to having only 12 clusters with 413 participants and could therefore not be used as a reliable single source for the sample size calculation.

We applied a Bayesian approach to modelling and estimation of the ICC for determination of the sample size using multiple ICC estimates from previously published studies. The modelling automatically accounted for uncertainty in the synthesised ICCs and produced an informative posterior ICC distribution which we used to determine the sample size. We describe here the process used and provide a how-to guide on the proposed framework to assist researchers in exploring the utility of this approach for their own trials.

## Methods

### Model

To specify the model for the ICC, we assume, similar to Turner et al.,^9^ that each estimate ${\hat{\rho }}_{m}$ (*m* = 1,…,*s*) follows a Normal distribution around its true value *ρ_m_*

$${\hat{\rho }}_{m}\sim N\left(\rho_{m},\mathrm{Var}({\hat{\rho }}_{m})\right)$$

For the distribution variance, we make use of Swiger et al.’s^12^ formula for estimating the asymptotic variance of the ICC estimate

$$\begin{array}{cc} \mathrm{Var}[{\hat{\rho }}_{m}] & =V(\rho_{m},N_{m},k_{m})\\ =\frac{2(N_{m}-1){\left(1-\;{\hat{\rho }}_{m}\right)}^{2}{\left(1+\left(\frac{N_{m}}{k_{m}\;}-1\right)\;{\hat{\rho }}_{m}\right)}^{2}}{{\left(\frac{N_{m}}{k_{m}}\right)}^{2}(N_{m}-k_{m})(k_{m}-1)} \end{array}$$

where *N_m_* is the total number of participants in the trial, and *k_m_* is the number of clusters. Swiger’s formula requires minimal information for calculating the ICC variance estimate and has been used in a number of methodological papers.^1^ Turner et al.^9^ have also followed this method. Moreover, Swiger’s formula has been compared with other methods for calculating variance, and it was concluded that all approaches lead to similar practical conclusions.^7^

### Eliciting information for Bayesian hierarchical ICC modelling

Although a single robust ICC estimate was not available for the planned cluster-randomised trial, there were a number of studies available with ICC estimates that had varying degrees of relevance, both to the planned trial population and its primary outcome. To identify a set of ICC estimates relevant to our setting, we conducted a structured literature search. To identify the studies, the trial team utilised the search strategy implemented in a paper by Sutton et al.^13^ reviewing the use and reporting of cluster-randomised trials in stroke. We conducted an electronic search of titles and abstracts in the PUBMED database, and titles, abstracts or keywords in the CENTRAL (Cochrane Trials) database to June 2014 using the search terms ‘trial’ AND ‘stroke’ AND (cluster-randomised OR group-randomised OR cluster randomised OR group randomised), to identify full papers reporting cluster-randomised trials of stroke-related interventions. Studies were included only if they reported estimated ICCs. Through the search, 16 studies relevant to the planned trial were identified, including the ICONS feasibility trial and 8 trials from the review by Sutton et al.^13^ Most of the selected trials evaluated interventions designed to reduce stroke incidence or improve stroke care; two of the trials were concerned with incontinence problems. A total of 34 ICC estimates were extracted, with some studies providing multiple ICCs. A summary of the data extracted from the selected studies is presented by Table 1. Supplemental Table 2 shows characteristics of the studies and ICCs.

**Table 1.** Characteristics of studies with available relevant ICC estimates included in Bayesian modelling.

Study	Source	Intervention	Study population	Outcome	ICC estimate	Number of patients	Number of clusters
1	Thomas et al.^14^	Systematic voiding programme.	Stroke patients with incontinence (UK).	Absence of incontinence at 12 weeks post-stroke.	0.00	413	12
2	Tannenbaum et al.^15^	Three experimental continence interventions: (1) continence education; (2) evidence-based self-management; (3) combined continence education and self-management.	Women aged 60 years and older with untreated incontinence (UK).	Patient’s global impression of improvement in continence questionnaire (PGI-I) measured at 3 months post-intervention.	0.05	259	71
3	Sackley et al.^16^	Staff education on continence care and mobility care and mobility training.	UK care home residents.	Rivermead Mobility Index at baseline and 6 weeks post-intervention.	0.37	34	6
4	Sackley et al.^17^	Occupational therapy provided to individuals and carer education.	UK care homes residents with moderate to severe stroke-related disability (Barthel Index (BI) score 4–15) except those with acute illness and those admitted for end-of-life care.	BI score at baseline.	0.26	173	12
				BI change to 3 months.	0.18		
				BI change to 6 months.	0.2		
				Global poor outcome at 3 months.	0.14		
				Global poor outcome at 6 months.	0.09		
5	Weir et al.^18^	Computer-based decision support system to aid selection of long-term antithrombotic therapy.	UK hospital in-patients or out-patients with a clinical diagnosis of acute ischaemic stroke or TIA; first investigation of an event occurring within preceding 4 months.	Change in relative risk of ischaemic and haemorrhagic vascular events relative to the option of ‘no antiplatelet or anticoagulant therapy’.	0.15	1952	16
6	De Luca et al.^19^	The intervention group staff (physicians, nurses and drivers) training on and delivery of evidence-based prehospital emergency clinical pathway based on experiential learning approach. The training was focused on teaching the personnel to identify stroke symptoms.	People living in the community aged <80 years (Italy, acute care/ community).	The proportion of eligible acute stroke patients correctly referred to stroke unit.	0.05	4895	20
7	Dirks et al.^20^	Intervention to increase thrombolysis rates by creating local stroke teams, identifying barriers to thrombolysis delivery, setting goals and planned actions, and updating acute stroke guidelines.	Patients ≥18 years with acute stroke who were admitted to the hospital within 24 h from onset of symptoms (Netherlands, acute care/community).	Treatment with rtPA (recombinant tissue Plasminogen Activator).	0.0154	5515	12
8	Johnston et al.^21^	Quality improvement in ischaemic stroke discharge orders comprising statin prescription; antihypertensive medication for those with hypertension; warfarin for all patients with atrial fibrillation (AF), except those with contraindication.	At least 40 years old, were the Kaiser Permanente Medical Care Plan (KPMCP) members with pharmacy benefits, and had been discharged alive to home or to a facility other than hospice (USA hospitals).	Composite binary variable comprising optimal treatment via all of: (1) documentation of filled statin prescription 6 m post-discharge; (2) achievement of controlled blood pressure 4–8 m post-discharge; (3) for those with AF, either documentation of a filled prescription for warfarin or an International Normalised Ratio blood test 6 m post-discharge or a contraindication to warfarin.	0.0038	3361	12
9	Jones et al.^22^	All nurses and health-care assistants on the participating stroke intervention units received a group teaching package to improve their understanding and clinical practice in patient positioning.	Patients on stroke rehabilitation units (UK hospitals): with stroke, dependent on another person to position limbs, with inability to move from sitting to standing without assistance.	Rivermead Mobility Index at 6 months post-stroke.	0.00	120	10
10	Lakshminarayan et al.^23^	(1) Audit and written feedback of baseline performance; (2) analysis of structural and knowledge barriers to stroke care identified by provider questionnaires; (3) use of clinical opinion leaders to deliver customised feedback to care providers; (4) use of hospital management leaders to overcome identified barriers to stroke care.	Stroke patients aged 30–84 years admitted through emergency room (US hospitals).	Three outcomes with associated ICCs, each is related to the provision of 3 or 4 indicators of quality of care:		2305	19
				acute care indicators.	0.005		
				inpatient care indicators.	0.004		
				discharge indicators.	0.0007		
11	McAlister et al.^24^	Educational lecture to patients with nonvalvular AF on reducing stroke risk, with self-administered booklet and individualised audiotape decision aid tailored to their personal stroke risk profile.	Adult patients with nonvalvular AF not living in institutions (Canada, Primary Care Practices).	Change in proportion of patients taking antithrombotic therapy appropriate to their stroke risk 3 months post-intervention.	0.02	434	102
12	Forster et al.^25^	Structured training programme for caregivers (the London Stroke Carers Training Course).	Patients at UK stroke units with a diagnosis of stroke, likely to return home with residual disability and with a caregiver providing support.	Self-reported extended activities of daily living at 6 months measured with the Nottingham Extended Activities of Daily Living scale.	0.027	928	36
13	Taylor et al.^26^	Structured goal elicitation using the Canadian Occupational Performance Measure.	Stroke patients admitted to inpatient rehabilitation services (New Zealand) with ‘sufficient’ cognition for goal setting and completing outcome assessment.	Quality of life at 12 weeks measured using the following tools:		41	4
				Schedule for Individualised Quality of Life (SEIQOL-DW).	0.40		
				The Medical Outcomes Study 36-item Short Form Health Survey (SF-36), Physical Component Summary (PCS) score.	0.24		
				Functional Independence Measure.	0.21		
				The Medical Outcomes Study 36-item Short Form Health Survey (SF-36), Mental Component Summary (MCS) score.	0.25		
14	Middleton et al.^27^	Treatment protocols to manage fever, hyperglycaemia and swallowing dysfunction with multi-disciplinary team building workshops to address implementation barriers.	Patients aged 18 years or older, who had a diagnosis of ischaemic stroke or intracerebral haemorrhage, and presented within 48 h of onset of symptoms to a participating Acute Stroke Unit (ASU), Australia.	Death and dependency 90 days after hospital admission.	0.018	1696	19
				Functional dependency BI ≥95, 90 days after hospital admission.	0.015		
				Functional dependency BI ≥60, 90 days after hospital admission.	0.009		
				SF-36 PCS score, 90 days after hospital admission.	0.026		
				SF-36 MCS score, 90 days after hospital admission.	0.011		
				Mean temp within 72 h in ASU.	0.084		
				At least one temperature ≥37.5°C in first 72 h.	0.009		
				Mean glucose during first 72 h in ASU.	0.056		
				Swallowing screening within 24 h of admission to ASU.	0.156		
15	Power et al.^28^	Stroke 90/10, a quality improvement collaborative based on the Breakthrough Series model.	Patients admitted to stroke units at NHS hospital Trusts in the Northwest of England.	Compliance with two evidence-based bundles of care: early hours and rehabilitation.		6592	24
				Early hours bundle.	0.066		
				Rehabilitation bundle.	0.197		
16	Dregan et al.^29^	Remotely installed electronic decision support tools to promote intensive secondary prevention.	Patients ever diagnosed with acute stroke (Family Practices, UK).	Systolic blood pressure.	0.032	11,391	106

ICC: intracluster correlation coefficient; TIA: transient ischemic attack; NHS: National Health Service.

To combine all 34 ICCs, we used the following model suggested by Turner et al.^9^

$$\begin{array}{c} {\hat{\rho }}_{\mathrm{ml}}\sim N\left(\rho_{\mathrm{ml}},V\left(\rho_{\mathrm{ml}},N_{\mathrm{ml}},k_{\mathrm{ml}}\right)\right)\\ \log\mathrm{it}\left(\rho_{\mathrm{ml}}\right)\sim N\left(\mu_{m},\frac{\sigma_{w}^{2}}{w_{\mathrm{ml}}}\right)\\ \mu_{m}\sim N\left(\mu ,\frac{\sigma_{b}^{2}}{w_{m}}\right) \end{array}$$

here, *ρ_ml_* is ICC for *l*th outcome within the *m*th study, *N_ml_* and *k_ml_* are the corresponding number of participants and the number of clusters, $\sigma_{w}^{2}$ and $\sigma_{b}^{2}$ are the within- and between-study variances, *w_m_* and *w_ml_* are the study and outcome weights, *m* = 1,…, *r*; *l* = *s*_1_,…,*s_m_*. In this model, the exchangeability is implied for both between and within studies so that the parameters may be considered as independently drawn from a common distribution.^8^ In the context of our model, exchangeability within each of the separate studies means that the estimates ${\hat{\rho }}_{\mathrm{ml}}$ are distributed around an underlying value *ρ_ml_*, with the *ρ_ml_* assumed exchangeable within studies and Normally distributed on the logit-transformed scale around a study-specific mean *μ_m_*. Logit transformation accounts for the permissible range of values [0, 1] for the *ρ_ml_*. Exchangeability between studies means that the *μ_m_* are assumed to be independently drawn from a common Normal distribution.

Similar to Turner et al.,^9^ we adjust for the varying relevance of the estimates by assigning weights to each study and to each outcome with an ICC. Study weights represent the degree of relevance to the study population and intervention; outcome weights reflect the degree of relevance to the planned trial’s primary outcome. By assigning a lower weight, we decrease the influence that the corresponding estimate has on the constructed posterior ICC distribution.

In practice, however, it is unclear how to assign these weights. Guidance^9^ suggests that relevance weights should express a proportion of the total nonsampling variance that is not due to bias. This advice is difficult to implement in practice, and the authors emphasise that these weights are likely to be subjective.

To minimise subjectivity in defining weights, we considered using a knowledge elicitation process to derive study and outcome weights from expert prior opinion or beliefs. We performed a ranking exercise where eight members of the trial team, with relevant expertise, assigned weights for each trial and for each outcome. The expert team included investigators with the following expertise: health-service and stroke researchers, nurses, methodologists and a stroke-survivor (member of ICONS Patient and Public Involvement group).

To aid elicitation, we produced an information leaflet (see Supplemental Material) explaining the methodology. An Excel spreadsheet (Supplemental Table 1) was developed summarising the 16 relevant studies. It was carefully designed to focus the expert elicitation process on the key parameters required for comparing studies and assigning weights, and to avoid creating a burdensome exercise for the reviewers.

The eight experts were tasked with assigning study and outcome weights. The ranking exercise was set up in two stages. The first stage comprised a group exercise and was conducted face-to-face, in line with methodological developments suggesting that elicitation within group meetings is an efficient way of collecting expert opinion.^30^ Reviewers were provided with an explanation of the method. Each expert was given an Excel spreadsheet with a summary of the extracted studies. The group was given an opportunity to work together on reviewing a few studies, with some interaction between experts. With the assistance of a project statistician facilitating the meeting, the experts discussed their initial estimates with the others to clarify the ranking exercise and establish a shared understanding of how to measure a relevance of outcome and study to the target setting. It has been shown that this first discussion round with interaction between experts is beneficial for the knowledge elicitation process as it generates sharing of knowledge and leads to improvements in response accuracy.^31^ At the second stage, the reviewers were asked to revise their ratings in light of the first round, rank all studies in their own time and return the individual judgements to a facilitator by email.

The implemented knowledge elicitation process is a version of the probabilistic Delphi protocol^32^ recommended by the European Food Safety Authority^33^ for work with multiple experts. It requires aggregation across the experts’ final estimates; this is described in the following section.

### Synthesising expert opinion

**Figure 1.**
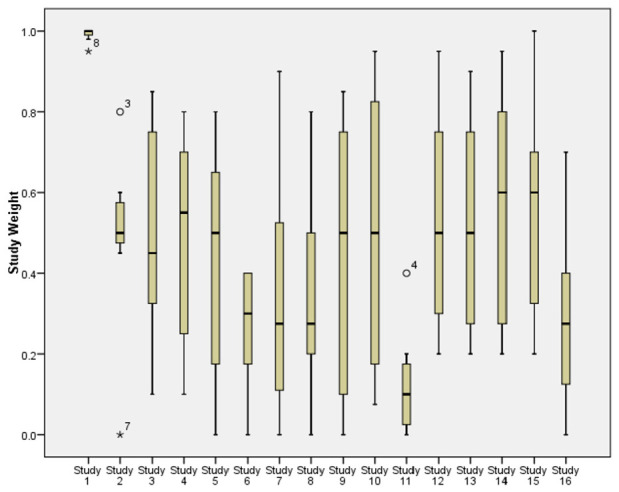
Boxplots showing the spread of reviewer responses about each study’s weight. Study numbers are in same order as in Table 1. The narrowest boxplot relates to the ICONS feasibility trial.

There was considerable diversity across the experts in the weights provided, with some reviewers having a tendency to assign much higher or much lower weights than others (Figure 1). The reviewers tended to be in better agreement for studies of lower relevance to the target study: the spread of responses was narrower for these studies when compared to the studies of higher relevance.

Before putting weights into the model, we assessed the reviewers’ performance in a series of steps described in the Supplemental Material, by checking agreement and reliability of the responses focussing on study weights. We measured inter-rater agreement between eight reviewers *R_i_*, *i* = 1, …, 8, and employed Reliability Analysis tools calculating Cronbach’s alpha coefficient and inter-item correlations matrix. This analysis suggested that two reviewers (*R*_4_ and *R*_5_) had poor performance-related characteristics and therefore should be treated differently to other raters.

To produce a collective study weight and outcome weight to put into the Bayesian model, we followed the approach of mathematical aggregation^32^ where separate judgements elicited from the experts are combined into the aggregate estimate using a pooling rule. We used linear opinion pooling. To reflect differences in their reliability, each reviewer was assigned a certain weight, called an importance weight, which controlled the input of each reviewer into the pooled opinion. We employed the Rank Sum weight method.^34^ In this approach, the weights are calculated as the individual ranks divided by the sum of the ranks:

$$\pi_{j}=\frac{\left(8-\mathrm{Ran}k_{j}\;+1\right)}{\mathrm{Sum}(8-\mathrm{Ran}k_{j}+1)}$$

where Rank_*j*_ is the rank of the *j*th reviewer, *j* = 1,…, 8, producing normalised weights summing to one. The elicitation evaluation process and sensitivity analysis described below and in the Supplemental Materials suggested that downgrading the input of *R*_4_ and *R*_5_ would improve quality of the elicited weights. We therefore assigned *R*_4_ and *R*_5_ the lowest rank 8, with all others assigned rank 1. This calibration process produced lower importance weights *π*_4_ = *π*_5_ = 0.02 to reduce the impact of reviewers *R*_4_ and *R*_5_ on the pooled study and outcome weights, and *π_j_* = 0.16 for all other reviewers. These importance weights were used in the modelling.

### Bayesian hierarchical ICC modelling

All 34 ICC estimates from Table 1 were combined in the Bayesian hierarchical model (Table 2).

**Table 2.** Bayesian hierarchical model specification.

Parameter	Proposal
${\hat{\rho }}_{\mathrm{ml}}$	*N* (*ρ_ml_*, *V*(*ρ_ml_*, *N_ml_*, *k_ml_*))
logit (*ρ_ml_*)	*N* (*μ_m_*, * $\sigma_{w}^{2}$ /w_ml_*)
*μ_m_*	*N* (*μ*, * $\sigma_{b}^{2}$ /w_m_*)
*μ*	*N* (0, 10 000)
*σ_w_*	*U* [0, 5]
*σ_b_*	*U* [0, 5]

*N_ml_* and *k_ml_* are the cluster size and number of clusters; vague priors *N* (0, 10 000) were assigned to mean *µ* for each model throughout; *U* [0, 5] is a uniform distribution.

**Figure 2.**
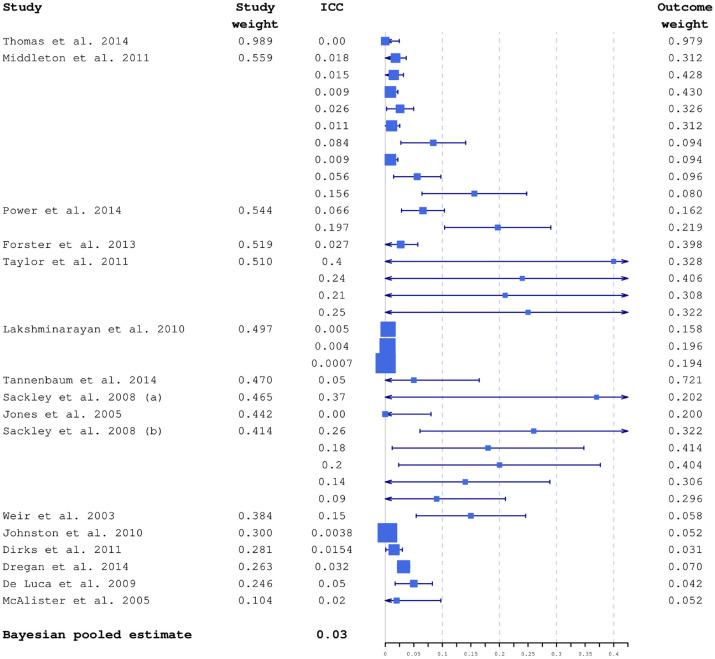
ICC estimates included in the modelling plotted together with 95% confidence intervals and average study and outcome weights. Box sizes are inversely proportional to variances. The studies are ordered by decreasing relevance to the planned study, based on estimated average study weight. The largest weights were from the ICONS feasibility trial.

Studies included in the modelling had considerable variation in sample sizes, and consequently in ICC precision. In Figure 2, all 34 ICCs are plotted together with their 95% confidence intervals calculated using Swiger’s formula.^35^ Characteristics of the extracted weights are also shown in Supplemental Table 2.

The models were fitted within WinBUGS.^36^

## Results of Bayesian ICC modelling

### Posterior ICC distribution

The constructed ICC distribution is summarised in Table 3. For the purpose of the sample size estimation, the ICC point estimate can be chosen by summarising the posterior distribution. The posterior median is the summary of location commonly used in practice.^8^ The 95% credible interval provides guidance regarding the probability of actually observing this particular ICC value and the adequacy of the planned sample size. For comparison, in a classical framework, the ICC estimate derived using the 34 ICCs from the identified studies could be calculated using one of the most commonly used approaches: median 0.05, mean 0.098, weighted mean 0.103 (using our outcome weights), maximum 0.4. These simple approaches do not take into account any differences between studies and their varying relevance to our target trial. Note that the maximum (0.4) is outside the 95% credible interval (0.00131–0.330) and is also likely to be overly conservative.

**Table 3.** Summaries of posterior distributions constructed for the ICC and the model standard deviations, between-study *S_b_* and within-study *S_w_*.

Variable	Posterior mean (SD)	MC error	2.5% percentile	25% percentile	Posterior median	75% percentile	97.5% percentile
ICC	0.0607 (0.0937)	0.000145	0.00131	0.012	0.0296	0.0682	0.330
*S_b_*	1.224 (0.409)	0.00369	0.633	0.940	1.156	1.43	2.217
*S_w_*	0.345 (0.0874)	0.000413	0.206	0.284	0.335	0.395	0.546

ICC: intracluster correlation coefficient; SD: standard deviation; MC: Monte Carlo.

### Sample size estimation

Modelling the ICC within a Bayesian framework provides the researcher with a full posterior distribution which can be summarised in a number of ways to provide the estimate for sample size calculation. We used the posterior median, although a range of posterior quantiles can be considered when designing a trial. For the post-stroke incontinence cluster-randomised trial, the sample size was chosen to provide at least 80% power with a 5% significance level to detect a minimally important between-group difference of 2.52 points^37^ in mean ICIQ-UI-SF 3-month score, using an independent-samples *t*-test and a common standard deviation 8.32 computed from data collected for the ICONS feasibility trial.^11^ An ICC was assumed to be less than or equal to the posterior median $\hat{\rho }=0.0296$ . The sample size was determined using PASS-16 sample size software.^38^ It was assessed as realistic to recruit between 40 and 50 stroke units which requires the total sample size of *N* = 480 and *N* = 450 for *k* = 20 and *k* = 25 clusters per arm, with an average sample size per cluster *m* = 12 and *m* = 9, respectively. The power was 82%.

The advantage of using a Bayesian approach to determine sample size is that it provides a method which allows for imprecision in the ICC estimate, and informs researchers designing trials about the range of plausible sample size values, as opposed to the standard approach where sample size is calculated as a single number using a point ICC estimate. With a constructed posterior distribution of ICC, trials can be designed using a range of plausible ICC values, such as quantiles within the 95% credible interval; using values from the upper range of the credible interval would, however, probably be too conservative.

**Figure 3.**
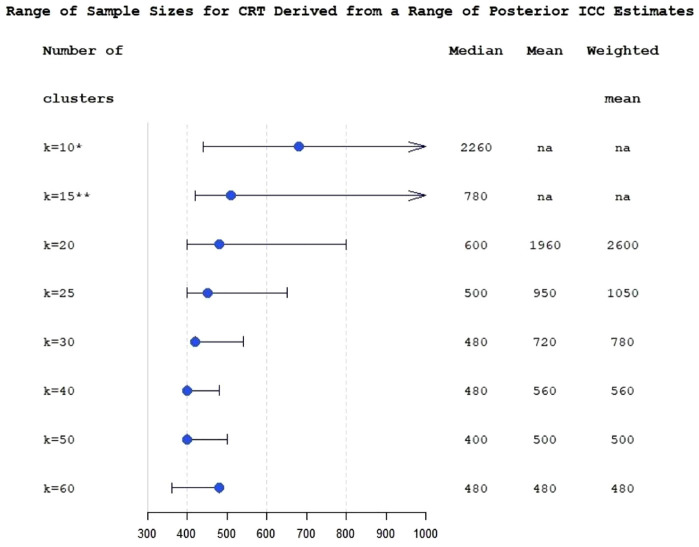
Range of sample sizes derived for different ICC values from posterior interquartile range of ICC estimate for the varying number of clusters at fixed levels (*k* from 10 to 60), for cluster-randomised trial with *k* equal size clusters per arm. The bullets are sample sizes calculated using posterior median ICC. The whiskers correspond to 25% and 75% posterior ICC quantiles. Median, mean and weighted mean columns show sample sizes calculated using a classical multi-estimate method. All numbers correspond to at least 80% power achieved; *: 80% power is not achievable for ICC 75% quantile; **: sample size corresponding to ICC 75% quantile is 1440; na: 80% power is not achievable for this number of clusters.

To incorporate the uncertainty about ICC into the sample size calculation, we used the posterior interquartile range of ICC to evaluate the range of plausible values of sample sizes which could be anticipated for the planned trial (Figure 3). The figure also shows sample sizes which would be obtained under the classical approach.

Figure 3 demonstrates that in this study the advantages of applying Bayesian modelling for ICC estimation are less apparent with large numbers of clusters. At *k* = 60, the sample sizes provided by the classical approach are the same as the sample size corresponding to the posterior median ICC (*N* = 480) due to the small number of subjects per cluster. Figure 3 also illustrates the problem with low and moderate numbers of clusters in randomised controlled trials (RCTs): sample size becomes acceptable for practical purposes and reasonably stable only when the number of clusters is around 25 or greater.

### Sensitivity analysis

To evaluate the sensitivity of the estimated ICC and sample size estimates to the model inputs and assumptions, we investigated sensitivity to (1) the choice of reviewers (and subsequently weights included in the modelling) by comparing three alternative versions of importance weights; (2) the choice of studies to be included into the modelling by investigating how focussing on most relevant studies (top 25%, 50% and 75%) would affect the results. Further details are in the Supplemental Material.

Sensitivity analysis demonstrated that the findings presented, and posterior ICC distribution in particular, showed limited sensitivity to the choice of the importance weights *π_j_* and that the model fit is worse in scenarios where only the most relevant trials were included in the model (see Supplemental Table 3) suggesting that it is better to be overinclusive in terms of potential relevance. The model implemented with two-category Rank Sum importance weights and all 16 studies included demonstrated better fit.

## Discussion

In this study, we employed a Bayesian framework that provides a flexible and informative way to handle ICC uncertainty and uses previously published or external ICC estimates. We have presented an extension to the approach proposed by Turner et al.^9^ and described the implementation of a method to construct posterior distribution of the ICC using external information from available ICCs and expert knowledge.

The method suggested by Turner et al.^9^ has been used in several studies and practical applications,^39,40^ and has been extended to count data.^41^ However, it has not yet been adopted widely. The approach does require knowledge of a relatively advanced Bayesian technique, but another obstacle is the uncertainty in choosing study and outcome weights. The methodology we have proposed helps to overcome this problem. Using expert knowledge reduces subjectivity in choosing weights and improves informativity and robustness of the ICC estimate.

With the conventional approach, when an ICC estimate is imprecise or unreliable, researchers tend to choose a conservative ICC for their sample size calculation.^1,42,43^ This often leads to an unnecessarily inefficient trial, with more clusters than strictly necessary and hence greater overall trial costs. The strength of the suggested approach is that it provides justification for a robust and typically smaller ICC compared to the conventional approach, leading to sample size reductions and thus resulting in substantial efficiency savings for the proposed trial.

Using complex Bayesian models may require a greater investment of time and expertise. However, the benefit of using a Bayesian model to estimate the trial ICC is that it offers greater flexibility for combining available ICC estimates while incorporating uncertainty and information about the relevance of these estimates into the model.

As practical guidance, researchers wishing to explore the utility of the proposed approach would need to consider the following steps:

1. Identify and select ICCs for relevant outcome measures from previous relevant studies, through systematic review and exploring existing databases. The recommendation is to be highly inclusive in terms of potential relevance.
2. Summarise the selected studies with existing ICCs in a Summary Table similar to Supplemental Table 1.
3. Identify and invite experts in the topic relevant to the project, agree on elicitation technique, conduct training and task them with a ranking exercise to assign weights *w_m_* to each study and *w_ml_* to each outcome within the study using the Summary Table.
4. As an optional step, we recommend considering evaluation of the expert elicitation and agreement, and differentiate expert input into the model by introducing importance weights *π_j_*, if required.
5. Aggregate the elicited weights and embed them into the Bayesian modelling of the targeted ICC.
6. Examine the sensitivity of the conclusions to the chosen model.
7. Choose an appropriate ICC estimate using the Bayesian posterior distribution of the ICC, and then use that to provide one or more estimates of the sample size.

The main strength of this study is that we have proposed a practical method of implementing the synthesis of externally available ICCs within a Bayesian framework using expert opinion.

The limitation of our approach is the validity and consistency of the reviewers’ ratings. This can be mitigated by increasing the number of reviewers, conducting more targeted training prior to the ranking exercise, and applying different knowledge elicitation techniques. As an extension, strategies for evaluating the elicitation exercise (including ranking reviewers, agreement and coherence checks, calibration)^30^ can be embedded within the elicitation process, together with examining sensitivity of the conclusions to the used models.^44^ A more advanced approach would be to set up calibration questions, where experts are asked questions where the truth is known.^45^ The choice of the reviewers in this study was a convenience purposive expert sample, and it could be improved in further practical implementation of the method.

Evaluating the elicited beliefs can be affected by a range of biases.^30,46^ Johnson et al.^46^ developed a conceptual framework outlining the belief-elicitation process. They emphasised that elicitation methods should be evaluated in respect to such measurement properties as *validity, reliability, responsiveness* and *feasibility*, with *validity* and *reliability* being a prerequisite for meeting methodological standards. For this study, evaluation of *validity* was limited as there was no gold standard for the elicitation of the required probability weights. *Reliability*, and inter-rater reliability in particular, was evaluated using appropriate measures of association. The *responsiveness* was not applicable in this study and the property of *feasibility* was not directly evaluated, although the elicitation process was designed to minimise required time, costs and need for equipment. Further research on developing methodological strategies to evaluate measurement properties would help to reduce the influence of potential biases on the weight elicitation in the proposed framework.

The methodology described in this article is proposed in the context of continuous outcome data, as was Swiger’s approach. However, Swiger’s formula can easily be extended to construct interval estimates for the ICC in the setting of binary outcome clustering by replacing the appropriate quantities in the formula with the binary outcome equivalents,^7^ expanding the practical applicability of the method.

The proposed methodology reduces the impact of uncertainty in the ICC on the design. A next step towards more robust study design could be calculating a mean power (assurance)^47–49^ using the ICC distribution produced by our method. Choosing a sample size to achieve a desired assurance, rather than to achieve a desired power, conditional on an assumed point estimate of the ICC, would help to protect further against loss of power, although the produced sample size would be typically larger.^50^

There are a range of knowledge elicitation tools and techniques which can influence effectiveness of the elicitation. Using online graphical elicitation tool can provide an accessible and intuitive framework for eliciting the information and would naturally produce prior distributions for statistical models.^44^ Applying leading elicitation protocols – such as Cooke, SHELF or probabilistic Delphi – can minimise bias and improve accuracy in multiple expert judgements.^32,33^ Future implementation of the method could be expanded to use elicitation software, such as the Sheffield Elicitation Framework and associated web-based version,^51,52^ to gather expert knowledge in the form of probability distributions for unknown quantities.

## Supplemental Material

sj-docx-1-ctj-10.1177_17407745231164569 – Supplemental material for Determining the sample size for a cluster-randomised trial using knowledge elicitation: Bayesian hierarchical modelling of the intracluster correlation coefficient Click here for additional data file. Supplemental material, sj-docx-1-ctj-10.1177_17407745231164569 for Determining the sample size for a cluster-randomised trial using knowledge elicitation: Bayesian hierarchical modelling of the intracluster correlation coefficient by Svetlana V Tishkovskaya, Chris J Sutton, Lois H Thomas and Caroline L Watkins in Clinical Trials

sj-docx-3-ctj-10.1177_17407745231164569 – Supplemental material for Determining the sample size for a cluster-randomised trial using knowledge elicitation: Bayesian hierarchical modelling of the intracluster correlation coefficient Click here for additional data file. Supplemental material, sj-docx-3-ctj-10.1177_17407745231164569 for Determining the sample size for a cluster-randomised trial using knowledge elicitation: Bayesian hierarchical modelling of the intracluster correlation coefficient by Svetlana V Tishkovskaya, Chris J Sutton, Lois H Thomas and Caroline L Watkins in Clinical Trials

sj-docx-4-ctj-10.1177_17407745231164569 – Supplemental material for Determining the sample size for a cluster-randomised trial using knowledge elicitation: Bayesian hierarchical modelling of the intracluster correlation coefficient Click here for additional data file. Supplemental material, sj-docx-4-ctj-10.1177_17407745231164569 for Determining the sample size for a cluster-randomised trial using knowledge elicitation: Bayesian hierarchical modelling of the intracluster correlation coefficient by Svetlana V Tishkovskaya, Chris J Sutton, Lois H Thomas and Caroline L Watkins in Clinical Trials

sj-docx-5-ctj-10.1177_17407745231164569 – Supplemental material for Determining the sample size for a cluster-randomised trial using knowledge elicitation: Bayesian hierarchical modelling of the intracluster correlation coefficient Click here for additional data file. Supplemental material, sj-docx-5-ctj-10.1177_17407745231164569 for Determining the sample size for a cluster-randomised trial using knowledge elicitation: Bayesian hierarchical modelling of the intracluster correlation coefficient by Svetlana V Tishkovskaya, Chris J Sutton, Lois H Thomas and Caroline L Watkins in Clinical Trials

sj-pdf-2-ctj-10.1177_17407745231164569 – Supplemental material for Determining the sample size for a cluster-randomised trial using knowledge elicitation: Bayesian hierarchical modelling of the intracluster correlation coefficient Click here for additional data file. Supplemental material, sj-pdf-2-ctj-10.1177_17407745231164569 for Determining the sample size for a cluster-randomised trial using knowledge elicitation: Bayesian hierarchical modelling of the intracluster correlation coefficient by Svetlana V Tishkovskaya, Chris J Sutton, Lois H Thomas and Caroline L Watkins in Clinical Trials
