# Supplementary material for: Determining the sample size for a cluster-randomised trial using knowledge elicitation: Bayesian hierarchical modelling of the intracluster correlation coefficient
Source: Clin Trials. 2023 Apr 10;20(3):293–306. doi: 10.1177/17407745231164569 (PMC10262340; doi:10.1177/17407745231164569)
Supplement: sj-docx-1-ctj-10.1177_17407745231164569 – Supplemental material for Determining the sample size for a cluster-randomised trial using knowledge elicitation: Bayesian hierarchical modelling of the intracluster correlation coefficient [file sj-docx-1-ctj-10.1177_17407745231164569.docx]

**Weights for ICC Estimation**

***Information for reviewers***

**Background**

The **Intracluster Correlation Coefficient** (ICC) plays a crucial role in designing cluster randomised trials. It is a measure of similarity among subjects within the same cluster, which is calculated as a ratio of within-group variance and between-group variance. The ICC is an important part of the sample size calculation because sample size is very sensitive to small differences in the ICC.

A robust ICC estimate is not currently available to inform the planning of the proposed ICONS II cluster trial. However, there are a number of studies available with ICC estimates that have varying degrees of relevance. These multiple ICC estimates can be combined, using an appropriate statistical modelling technique, to produce a more targeted ICC estimate to support the determination of the sample size for ICONS II. In order to implement this modelling, some additional information is required from existing studies. This information will be based on the relevance of the studies (and their outcomes) to ICONS II and, specifically, to the primary outcome for ICONS II.

**Aim**

To rank the attached studies with regard to their relevance to the proposed primary outcome for the ICONS II cluster trial, namely “Severity of urinary incontinence (UI) at 3 months from baseline” measured by International Consultation on Incontinence Questionnaire – Urinary Incontinence Short Form (ICIQ-UI-SF) for those not catheterised. The ICIQ-UI-SF total score^10^ will be determined using the following algorithm:

*ICIQ-UI total score (0-21) = How often do you leak urine (0-5) + How much urine do you usually leak (0-6) + Overall, how much does leaking urine interfere with your daily life (0-10).*

Higher scores indicate greater severity in UI. Participants who are catheterised do not complete the ICIQ-UI and will be given a maximum ICIQ-UI score of 21.

**Method**

You will be required to assign two sets of **weights** to each of the attached studies. These weights should be based on the extent to which (i) the study and (ii) its outcome are relevant to the proposed ICONS II cluster trial and its proposed primary outcome variable. (These weights could be seen as ‘distances’ between the study / outcome and the ICONS II study / outcome.)

To assist with the ranking process, you have been provided with a table (in an Excel worksheet) that contains a list of the studies which we believe should help to inform the ICC (the studies have been identified from systematic searches of relevant databases) estimate for the primary outcome. In the table there is also some key information about each of these studies. You are required to complete two of the columns in the table: 'Study Weight' and 'Outcome Weight'

**Study Weight** is the degree of relevance (or closeness) of the ***study’s population*** to the ***study population of ICONS II***.

- The weights should represent the relevance of the study population. The characteristics to consider for study weight may include (but not be limited to): setting; type of cluster; general population; intervention. For example, if the setting was a stroke unit, with the cluster based on hospital, the sample included stroke patients, and the intervention was around repetitive task training, you might decide to weight that study quite highly because a number of the study’s characteristics are quite similar to those of ICONS II.
- The weight should be a percentage value in the range from 0 to 100% with 0% for completely irrelevant and 100% for a fully relevant study population.

**Outcome Weight** is the degree of relevance (closeness) of ***study outcome*** to the ***ICONS II study’s primary outcome***, the ICIQ-UI-SF total score.

There may be several outcomes within the same study. You should only consider the outcomes that are listed in the table (they are the ones that we have identified as having an associated ICC). You will need to consider whether or not the outcomes are relevant to the ICONS II study’s primary outcome. The outcomes that would be relevant include: measures of urinary incontinence, incontinence-specific quality of life and activities of daily living measures containing one or more items relating to incontinence (e.g. Barthel). For example, if an outcome was based around urinary incontinence, then it would be highly relevant, while an outcome based around depression would have low relevance. A quality of life outcome would probably be of moderate relevance, depending on the contribution of the effects of incontinence to the quality of life measure (so the Incontinence Quality of Life measure would have greater relevance than the Euroqol [EQ-5D]).

- As with the study weight, the outcome weight should be a percentage value in the range from 0 to 100% with 0% for completely irrelevant and 100% for a fully relevant outcome.
