# Supplementary material for: Determining the sample size for a cluster-randomised trial using knowledge elicitation: Bayesian hierarchical modelling of the intracluster correlation coefficient
Source: Clin Trials. 2023 Apr 10;20(3):293–306. doi: 10.1177/17407745231164569 (PMC10262340; doi:10.1177/17407745231164569)
Supplement: sj-docx-3-ctj-10.1177_17407745231164569 – Supplemental material for Determining the sample size for a cluster-randomised trial using knowledge elicitation: Bayesian hierarchical modelling of the intracluster correlation coefficient [file sj-docx-3-ctj-10.1177_17407745231164569.docx]

**Supplementary Table 2. Characteristics of studies, extracted ICC estimates and assigned weights**

| **Parameter** | **Minimum** | **Maximum** | **Median** |
| --- | --- | --- | --- |
| ICC estimate | 0 | 0.40 | 0.05 |
| Number of patients in trial | 34 | 11391 | 670.5 |
| Number of clusters | 4 | 106 | 19 |
| Mean cluster size | 4 | 275 | 26 |
| Study weights (m=16) | 0.4438 | 0.9913 | 0.525 |
| Outcome weights (l=34) | 0.0844 | 0.9838 | 0.317 |
