# Supplementary material for: Determining the sample size for a cluster-randomised trial using knowledge elicitation: Bayesian hierarchical modelling of the intracluster correlation coefficient
Source: Clin Trials. 2023 Apr 10;20(3):293–306. doi: 10.1177/17407745231164569 (PMC10262340; doi:10.1177/17407745231164569)
Supplement: sj-docx-4-ctj-10.1177_17407745231164569 – Supplemental material for Determining the sample size for a cluster-randomised trial using knowledge elicitation: Bayesian hierarchical modelling of the intracluster correlation coefficient [file sj-docx-4-ctj-10.1177_17407745231164569.docx]

**Assessing Reviewers’ Performance**

**Steps applied to assess reviewers’ performance:**

1. Inter-rater agreement between eight reviewers, measured in SPSS as a single measure estimate of the absolute agreement ICC, was 0.32, 95% CI = (0.13, 0.59), which means a fair agreement according to Landis and Koch^53^.
2. All raters were reasonably consistent in their ratings with an ICC consistency measure of 0.59, 95% CI = (0.40, 0.79).
3. The reliability of reviewers in assessing the degree of relevance of each study to our target study was also checked using SPSS Reliability Analysis tools. Cronbach’s alpha coefficient was high at 0.92.
4. The means and standard deviations of each reviewer’s study ratings, for all reviewers R_i_, i=1, …, 8, were correspondingly: for R_1_ 0.61 (0.23), R_2_ 0.28 (0.21), R_3_ 0.61 (0.25), R_4_ 0.30 (0.26), R_5_ 0.80 (0.19), R_6_ 0.59 (0.20), R_7_ 0.21 (0.29), R_8_ 0.30 (0.22). The reviewers appear to form two sub-clusters with fairly similar mean responses within a cluster (three reviewers with means 0.6 and four reviewers with mean 0.3 or less), with the exception being Reviewer 5 whose mean response was much higher than others at 0.8.
5. The instability in Reviewer 5 performance noticed at the previous step was supported by SPSS inter-item correlations matrix showing how each reviewer correlates to the other reviewers. The mean pairwise correlation for each reviewer was given by (0.65, 0.64, 0.71, 0.57, 0.59, 0.71, 0.69, 0.66) with Reviewer 5 having one of the lowest mean correlations of 0.59. Another atypical reviewer identified from the inter-item correlations matrix was Reviewer 4, who had the lowest mean pairwise correlation of 0.57 and whose correlations with five other reviewers were below 0.5.
6. The reliability of the reviewers was also tested using their correlations with the overall study weight exercise. Reviewers 4 and 5 had the lowest correlations (0.61 and 0.64), while mean correlation for all raters was 0.74.
7. As the previous steps suggested that Reviewers 4 and 5 should potentially be removed from the ratings, we analysed the Cronbach’s alpha score if corresponding item (reviewer) was removed from the weighting exercise. The score was 0.92 for both R_4_ and R_5_ which means deleting these reviewers would not lead to an improvement in Cronbach's alpha though their performance can affect stability of the results of the rating exercise.

The reliability analysis led to the conclusion that Reviewers 4 and 5 should be treated differently to other raters. As a result, the input of Reviewers 4 and 5 was downgraded with process described in section ’Synthesising expert opinion’.
