# Supplementary material for: Determining the sample size for a cluster-randomised trial using knowledge elicitation: Bayesian hierarchical modelling of the intracluster correlation coefficient
Source: Clin Trials. 2023 Apr 10;20(3):293–306. doi: 10.1177/17407745231164569 (PMC10262340; doi:10.1177/17407745231164569)
Supplement: sj-docx-5-ctj-10.1177_17407745231164569 – Supplemental material for Determining the sample size for a cluster-randomised trial using knowledge elicitation: Bayesian hierarchical modelling of the intracluster correlation coefficient [file sj-docx-5-ctj-10.1177_17407745231164569.docx]

**Sensitivity Analysis**

The different scenarios and models are compared using posterior median ICC, corresponding credible interval, and Deviance Information Criteria (DIC) statistics which measures the models’ adequacy^54^.

**Sensitivity Analysis Scenario 1.**

To recognise uncertainty in the individual reviewers’ responses, and in how these responses were then pooled, we ran the model with alternative reviewers’ importance weights: (a) equal weights for all reviewers π_j_ = 0.125 and (b) Rank Sum approach weights calculated using Rank Sum weight formula given. With the former approach, experts are equally weighted so no expert is considered “better” than any other expert. For the latter set of weights, we made use of Cronbach’s alpha score recalculated if the item (reviewer) was removed provided by SPSS reliability analysis, and assigned ranks to each reviewer according to this score. This provided importance weights ranging from 0.18 to 0.068. The corresponding modelling results are in Supplementary Table 3 (CrI is credible interval).

Equal and differential input of the reviewers into the models’ study and outcome weights produce higher posterior ICC estimates and wider credible intervals compared to the main model results presented in the last row of the table. Rank Sum importance weights demonstrate better fit based on DIC value.

**Sensitivity Analysis Scenario 2.**

The studies included in the Bayesian modelling have a wide span of relevance to our target study, with study weights ranging from 0.104 to 0.989. We investigated how focussing on most relevant studies would affect the results and re-ran the analysis for the top 25%, 50% and 75% most relevant studies. Supplementary Table 3 shows that including only most relevant trials in the model produced a lower ICC when estimated as posterior median (the difference is less pronounced for the mean). However, model fit as assessed by DIC is worse in such scenarios, and also the credible interval becomes very wide, substantially adding uncertainty about the estimated ICC.

**Supplementary Table 3. Sensitivity analysis modelling results.**

| **Change in the model** | **Posterior median ICC (95% CrI)** | **CrI width** | **DIC*** |
| --- | --- | --- | --- |
| Equal importance weights  π_j_ = 0.125, j = 1, …, 8 | 0.0309 (0.00123, 0.354) | 0.353 | -135.3 |
| Rank Sum importance weights | 0.0304 (0.00119, 0.362) | 0.361 | -135.6 |
| 4 top relevant studies | 0.0196 (0.0000182, 0.746) | 0.745 | -62.6 |
| 8 top relevant studies | 0.0216 (0.0000589, 0.715) | 0.715 | -92.1 |
| 12 top relevant studies | 0.0283 (0.000351, 0.539) | 0.538 | -115.0 |
| 16 studies with Rank Sum importance weights (0.16 & 0.02) | 0.0296 (0.00131, 0.330) | 0.329 | -135.7 |

* Lower DIC demonstrates better fit.

The model implemented with two-category Rank Sum importance weights and all 16 studies included had the smallest DIC and the 95% credible interval had the smallest coverage of ICC values, thus providing a less uncertain and more robust ICC for use in designing the proposed trial.

The sensitivity analysis 2 leads to the recommendation that it is better to be over-inclusive in terms of potential relevance of the studies as this will be accounted for in the modelling.
