# Supplementary material for: Determining the sample size for a cluster-randomised trial using knowledge elicitation: Bayesian hierarchical modelling of the intracluster correlation coefficient
Source: Clin Trials. 2023 Apr 10;20(3):293–306. doi: 10.1177/17407745231164569 (PMC10262340; doi:10.1177/17407745231164569)
Supplement: sj-pdf-2-ctj-10.1177_17407745231164569 – Supplemental material for Determining the sample size for a cluster-randomised trial using knowledge elicitation: Bayesian hierarchical modelling of the intracluster correlation coefficient [file sj-pdf-2-ctj-10.1177_17407745231164569.pdf]

Study / Source	Intervention	Setting	Level of Clustering	Study Population	Number of Patients in trial	Number of Clusters	Outcome	ICC estimate	R1 Outcome Weight	R1 Study weight	R2 Outcome Weight	R2 Study weight	R3 Outcome Weight	R3 Study weight	R4 Outcome Weight	R4 Study weight	R5 Outcome Weight	R5 Study weight	R6 Outcome Weight	R6 Study weight	R7 Outcome Weight	R7 Study weight	R8 Outcome Weight	R8 Study weight
		Primary or secondary care etc		Characteristics of patients recruited			Relevant outcomes with ICC estimate available																	
Thomas et al. 2014 ICONS: Identifying Continence OptioNs after Stroke	Systematic voiding programme	UK, Hospital	Stroke services		413 consented	12	Absence of incontinence (at 12 weeks post-stroke)	0.00	100	100	97.00	98.00	100	100.0	100	100.0	100	100.0	100	100.0	100	100	90	95
Tannenbaum et al. 2014 Effectiveness of continence promotion for older women via community organisations: a cluster randomised trial	Three experimental continence interventions: 1) continence education; 2) evidence-based self-management; 3) combined continence education and self-management.	UK, Community	Community organisations	Women aged 60 years and older with untreated incontinence	259 consented	71	ICC: Patient's global impression of improvement in continence questionnaire (PGI-I) measured at 3 months postintervention.	0.05	80	50	83.00	45.00	90	80.0	50	50.0	90	60.0	80	50.0	50	0	50	55
Sackley et al. 2008 A phase II exploratory cluster randomised controlled trial of a group mobility training and staff education intervention to promote urinary continence in care homes.	Staff education on continence care and mobility care and mobility training.	UK care homes	Care homes	Care home residents	34 consented	6	ICC: Rivermead Mobility Index at baseline and 6 weeks postintervention.	0.37	20	80	15.00	25.00	30	70.0	60	40.0	70	85.0	10	50.0	0	10	35	40
Sackley et al. 2008 Cluster randomized pilot controlled trial of an occupational therapy intervention for residents with stroke in UK care homes.	Occupational therapy provided to individuals and carer education.	UK, care homes	Care homes	All residents with moderate to severe stroke-related disability (BI score 4 to 15) were included except those with acute illness and those admitted for end-of-life care.	173 residents	12 care homes	Pilot trial with aim of estimating ICC for full trial; sample size based on Barthel Index. Five outcomes for weighting are below.	Intracluster correlation coefficients were high: 0.26 for Barthel score at baseline, 0.18 for Barthel change to 3 months, 0.20 for Barthel change to 6 months, 0.14 for global poor outcome at 3 months, 0.09 for global poor outcome at 6 months.		70		15.00		60.0		70.0		80.0		50.0		10		35
							Barthel score at baseline	0.26	20		22.50		40		70		0		30		50		30	
							Barthel change to 3 months	0.18	40		30.00		60		70		80		30		50		30	
							Barthel change to 6 months	0.2	40		27.50		55		70		90		30		50		30	
							Global poor outcome at 3 months	0.14	20		15.00		50		70		60		10		50		30	
							Global poor outcome at 6 months	0.09	20		12.50		45		70		70		10		50		30	
Weir 2003 (PRISM Study Group) Cluster-randomized, controlled trial of computer-based decision support for selecting long-term anti-thrombotic therapy after acute ischaemic stroke.	Computer based decision support system.	UK, secondary care	Hospital	In-patients or out-patients with a clinical diagnosis of acute ischaemic stroke or TIA; first investigation of an event occuring within preceding four months.	1952 patients	16 hospitals	ICC: relative risk reduction.	0.15	10	70	10.00	20.00	0	60.0	0	40.0	50	80.0	0	60.0	0	0	10	15
De Luca et al. 2009. An emergency clinical pathway for stroke patients – results of a cluster randomised trial	The EMS and ER health professionals (physicians, nurses and drivers) in the intervention group were trained to apply the ECP procedures using the educational method in line with the experiential learning tradition. The training was focused on teaching the personnel to identify stroke symptoms	Italy (Rome), Acute care/community	Emergency Medical Service (EMS) and Emergency Room (ER)	People living in the community aged <80	4895	20	The proportion of eligible acute stroke patients correctly referred to the SU*	0.05	10	40	5.00	25.00	0	30.0	0	30.0	10	40.0	0	40.0	0	0	10	10
Dirks et al. 2011. Promoting Thrombolysis in Acute Ischemic Stroke	Intervention meetings based on the Breakthrough Series model. Teams noted specific local barriers to further implementation of rtPA, to set goals, and to plan actions to reach these goals	Netherlands, Acute care/community	Hospital	Patients >18 years with acute stroke who were admitted to the hospital within 24 hours from onset of symptoms	5515	12	Treatment with rtPA	0.0154	10	30	5.00	25.00	0	45.0	0	20.0	20	90.0	0	60.0	0	0	2	2

Study / Source	Intervention	Setting	Level of Clustering	Study Population	Number of Patients in trial	Number of Clusters	Outcome	ICC estimate	R1 Outcome Weight	R1 Study weight	R2 Outcome Weight	R2 Study weight	R3 Outcome Weight	R3 Study weight	R4 Outcome Weight	R4 Study weight	R5 Outcome Weight	R5 Study weight	R6 Outcome Weight	R6 Study weight	R7 Outcome Weight	R7 Study weight	R8 Outcome Weight	R8 Study weight
		Primary or secondary care etc		Characteristics of patients recruited			Relevant outcomes with ICC estimate available																	
Johnston et al. 2010. Standardized Discharge Orders after Stroke: Results of the Quality Improvement in Stroke Prevention (QUISP) Cluster Randomized Trial	Quality improvement in ischemic stroke discharge orders comprising statin prescription; antihypertensive medication for those with hypertension; warfarin for all patients with AF, except those with contraindication.	California, USA. Secondary care	Hospital	At least 40 years old, were KPMCP members with pharmacy benefits, and had been discharged alive to home or to a facility other than hospice	3361	12	Composite binary variable comprising optimal treatment via all of: 1) Documentation of filled statin prescription 6 m postdischarge; 2) achievement of controlled blood pressure 4–8 m postdischarge; 3) For those with AF, either documentation of a filled prescription for warfarin or an International Normalized Ratio blood test 6 m postdischarge or a contraindication to warfarin	ICC estimated from study data: 0.0038	10	40	5.00	25.00	0	30.0	0	20.0	20	80.0	0	60.0	0	0	15	20
Jones et al. 2005. Effect of recommended positioning on stroke outcome at six months: a randomized controlled trial	All nursing staff on the intervention units received a group teaching package to improve their clinical practice in patient positioning	UK, secondary care (rehabilitation)	Hospital (Stroke Unit)	Patients on stroke rehabilitation units: stroke, dependent on another person to position limbs, inability to move from sitting to standing without assistance	120	10	RMI (6-months post-stroke)	0	20	85	15.00	30.00	60	70.0	0	10.0	80	80.0	10	70.0	0	0	10	10
Lakshminarayan et al. 2010. A cluster-randomized trial to improve stroke care in hospitals	1) audit and written feedback of baseline performance; 2) analysis of structural and knowledge barriers to stroke care identified by provider questionnaires; 3) use of clinical opinion leaders to deliver customized feedback to care providers; 4) use of hospital management leaders to overcome identified barriers to stroke care	Minnesota, USA. Secondary care	Hospital	Stroke patients aged 30–84 years admitted through ER	2305	19	There are 3 outcomes that have associated ICCs. Each is associated with the provision of 3 or 4 markers of quality of care. Three outcomes for weighting are below.			85		7.50		80.0		10.0		95.0		70.0		30		25
							Acute care – IV tPA#	0.005#	10.0		5.0		0.0		0.0		30.0		0.0		50.0		30.0	
							In-hospital care^	0.004^	20.0		5.0		0.0		0.0		60.0		10.0		50.0		30.0	
							Discharge care~	0.0007~	20.0		5.0		0.0		0.0		50.0		10.0		50.0		30.0	
McAlister et al. 2005. Impact of a patient decision aid on care among patients with nonvalvular atrial fibrillation: a cluster randomized trial	Self-administered booklet and audiotape decision aid tailored to their personal stroke risk profile.	Alberta, Canada. Primary care	Primary Care Practices	Adult patients with NVAF not living in institutions	434	102	Receiving appropriate care (Antithrombotic prescribing (3 months))	0.02	10.0	20.0	5.0	5.0	0.0	10.0	0.0	0.0	20.0	40.0	0.0	10.0	0.0	0.0	15.0	15.0
Forster et al. 2013 A structured programme for caregivers of impatients after stroke (TRACS): a cluster randomised controlled trial and cost-effectiveness analysis.	Structured training programme for caregivers (the London Stroke Carers Training Course)	UK: stroke units	Stroke unit	Patients with a diagnosis of stroke, likely to return home with residual disability and with a caregiver providing support.	928	36 stroke units	ICC calculated using primary outcome, self-reported extended activities of daily living at 6 months measured with the Nottingham Extended Activities of Daily Living scale.	ICC used to estimate sample size required: no greater than 0.05. ICC adjusted based on study findings: 0.027	60.0	80.0	30.0	30.0	60.0	70.0	0.0	20.0	70.0	95.0	10.0	70.0	50.0	30.0	30.0	30.0
Taylor et al. 2011. A pilot cluster randomized controlled trial of structured goal-setting following stroke	Structured goal elicitation using the Canadian Occupational Performance Measure	New Zealand: Inpatient rehabilitation facilities	Rehabilitation services	Stroke patients admitted to inpatient rehabilitation with 'sufficient' cognition for goal setting and completing outcome assessment	41	4	Outcomes at 12-weeks. (NB other ICCs available). Schedule for Evaluation of Individual Quality of Life. Four outcomes for weighting are below.			50.0		30.0		80.0		20.0		90.0		70.0		50.0		25.0
							Schedule for Evaluation of Individual Quality of Life (SEIQOL-DW)	0.40(0.05-0.88)#	10.0		40.0		50.0		0.0		80.0		10.0		50.0		35.0	
							Functional Independence Measure (FIM)	0.21(0.01-0.80)~	30.0		30.0		70.0		0.0		70.0		30.0		50.0		35.0	
							Medical Outcomes Study 36-item Short Form Health Survey (SF-36) Mental Component Summary Score	0.25(0.009-0.78)'	20.0		20.0		50.0		0.0		60.0		10.0		50.0		35.0	
							Medical Outcomes Study 36-item Short Form Health Survey (SF-36) Physical Component Summary Score	0.24(0.009-0.81)^	20.0		27.5		50.0		0.0		70.0		10.0		50.0		35.0	

Study / Source	Intervention	Setting<br>Primary or secondary care etc	Level of Clustering	Study Population<br>Characteristics of patients recruited	Number of Patients in trial	Number of Clusters	Outcome<br>Relevant outcomes with ICC estimate available	ICC estimate	R1 Outcome Weight	R1 Study weight	R2 Outcome Weight	R2 Study weight	R3 Outcome Weight	R3 Study weight	R4 Outcome Weight	R4 Study weight	R5 Outcome Weight	R5 Study weight	R6 Outcome Weight	R6 Study weight	R7 Outcome Weight	R7 Study weight	R8 Outcome Weight	R8 Study weight
Middleton et al. 2011. Implementation of evidence-based treatment protocols to manage fever, hyperglycaemia, and swallowing dysfunction in acute stroke (QASC): a cluster randomised controlled trial	Treatment protocols to manage fever, hyperglycaemia, and swallowing dysfunction with multidisciplinary team building workshops to address implementation barriers	Australia: Secondary care	Astute Stroke Units	Acute stroke patients >18, presented <48hrs	1696	19	Nine outcomes for weighting are below. Unclear if these are from this study or not:	0.018#<br>0.015^<br>0.009~<br>0.026'<br>0.011†<br>0.084‡<br>0.009¶<br>0.056^^<br>0.156~~		70.0		25.0		90.0		20.0		95.0		70.0		50.0		30.0
							Death and dependency#		20.0		10.0		60.0		0.0		80.0		10.0		50.0		35.0	
							Barthel ≥95^		40.0		27.5		75.0		0.0		80.0		30.0		50.0		35.0	
							Barthel ≥60~		40.0		27.5		75.0		0.0		90.0		30.0		50.0		35.0	
							SF-36 MCS'		20.0		27.5		50.0		0.0		90.0		10.0		50.0		35.0	
							SF-36 MCS†		20.0		20.0		50.0		0.0		80.0		10.0		50.0		35.0	
							Mean temp within 72hrs‡		10.0		12.5		0.0		0.0		10.0		0.0		0.0		35.0	
							One or more temp ≥37.5 in 1st 72hrs¶		10.0		11.0		0.0		0.0		20.0		0.0		0.0		35.0	
							Mean glucose with 72hrs^^		10.0		12.5		0.0		0.0		20.0		0.0		0.0		35.0	
							Swallow screen within 24hrs adm ASU~~		5.0		5.0		0.0		0.0		40.0		0.0		0.0		35.0	
Power et al. 2014 Did a quality improvement collaborative make stroke care better? A cluster randomized trial	Stroke 90/10, a quality improvement collaborative.	UK: Stroke units in NHS hospitals	NHS Hospital Trusts	Patients admitted to stroke units.	6592	24 Trusts	ICC estimated based on compliance with two evidence-based bundles of care: early hours and rehabilitation. Two outcomes for weighting are below.	ICC calculated from study data: Early hours bundle: 0.066; Rehabilitation bundle: 0.197		70.0		25.0		70.0		20.0		100.0		70.0		50.0		40.0
							Early hours bundle		10.0		5.0		0.0		0.0		50.0		10.0		30.0		40.0	
							Rehabilitation bundle		10.0		7.0		0.0		0.0		80.0		20.0		50.0		40.0	
Dregan et al. 2014 Point-of-care cluster randomized trial in stroke secondary prevention using electronic health records	Remotely installed electronic decision support tools to promote intensive secondary prevention.	UK: community	Family practice	Patients ever diagnosed with acute stroke	11,391	106	ICC calculated using systolic blood pressure.	ICC calculated from data: 0.032	10.0	40.0	12.5	20.0	0.0	30.0	0.0	5.0	10.0	70.0	0.0	40.0	0.0	0.0	20.0	25.0
